# Ecofriendly Palladium on Wool Nanocatalysts for Cyclohexene Hydrogenation

**DOI:** 10.3390/nano8080621

**Published:** 2018-08-15

**Authors:** Sedigheh Ghadamgahi, James H. Johnston, Carla Fonseca-Paris

**Affiliations:** 1 Department of Chemistry, East Tehran Branch, Islamic Azad University, Tehran, Iran; 2 School of Chemical and Physical Sciences, Victoria University of Wellington, Wellington 6140, New Zealand; Jim.Johnston@vuw.ac.nz (J.H.J.); carlitafp@yahoo.com (C.F.-P.)

**Keywords:** palladium nanoparticles, wool, hydrogenation, cyclohexene, catalysis

## Abstract

Use of natural wool fiber supports in the fabrication of novel composite materials incorporating metal nanoparticles, which offer the possibility of “environmentally friendly” catalytic materials, has been investigated. The catalytic hydrogenation of cyclohexene to cyclohexane by palladium nanoparticles immobilized on wool (Pd/wool) was studied using moderate pressure of pure hydrogen gas. The performance of wool-supported catalysts was explored over a palladium nanoparticle loading ranging from 1.6 to 2.6 wt %. The effect of the catalytic testing conditions, including stirring rate, amount of reactants, gas pressure, and target temperature were explored. A systematic series of catalytic-activity tests carried out at 400 psi H_2_ for 5 and 24 h reaction times at 40 °C using a stirring rate 750 rpm allowed us to identify differences in performance within the series of Pd/wool nanocatalysts studied. The most catalytically active samples contained Pd nanoparticles with average sizes of ca. 5 nm located predominantly on the surface and within the topmost layer of wool fibers, making them more accessible to the reactants.

## 1. Introduction

Wool fibers and also wool textiles offer interesting natural support material for capturing noble metal nanoparticles, notably palladium and gold, onto the surface to provide wool–nanoparticle composites for catalysis applications. This application distinguishes wool from synthetic fibers, as wool comprise keratin protein molecules in its structure, which have S and N entities in the amino acid components that can readily bind to noble metals, such as palladium, gold, and silver. The naturally porous structure of wool fibers can also facilitate hosting of such metal nanoparticles. Collectively, these attributes provide an interesting opportunity to create and demonstrate palladium nanoparticle–wool composite fiber and textile materials as potential catalysts wherein the wool fibers provide the natural substrate. This research is presented here using New Zealand merino and crossbred wool types.

The important role of catalysts in chemistry is well known. Around 85–90% of all industrial chemical processes worldwide use heterogeneous catalysts [1], creating an added value of US$24.1 billion in 2018 [2]. It has long been known that noble metals can act as active and selective catalysts in many applications, but their industrial utility as bulk materials is diminished by their high cost and low surface area [3]. Palladium is a well-known catalyst, even as a bulk-phase metal, but its high cost had driven the industry towards its use in nanoparticle-based systems well before nanotechnology attracted the attention of the media. Nanoparticulate materials can provide greater specific surface areas at much lower costs. Metal nanoparticles have increased chemical activity as a result of their nanoscale size, surface-to-volume ratios, and crystallographic surface structure. Intensive research on noble-metal nanoparticles has demonstrated their exceptional catalytic properties and has led to significant improvements in the design and fabrication of catalytic materials [4]. Over the last two decades, the use of platinum, ruthenium, and palladium nanoparticles has burgeoned, as these (and other metals) have become established as active and selective catalysts for a large number of reactions [3,5,6]. Studies of palladium have been particularly extensive due to its remarkable properties as a highly active and selective catalyst, particularly for the hydrogenation of unsaturated hydrocarbons, such as cyclohexene, hex-1-ene, styrene, acrylic acid, phenol, and nitrobenzene [7,8,9,10]. The nature of supports can affect catalytic activity and selectivity via tuning of the metal-support interactions. Increasing recognition of the environmental ramifications of industrial activity has motivated the development of environmentally benign chemical processes and methodologies using natural-support materials [9,11]. Natural supports that incorporate nanosized metal particles could possibly be suitable as environmentally friendly substrates for a new generation of heterogeneous catalysts, provided the support material is sufficiently robust to stand the operational conditions of the catalytic reaction. Lately, metal particles immobilized on natural, renewable, and environmentally friendly fibrous supports, such as cotton, animal hair, and silk have attracted the attention of researchers [11,12,13]. Nanosized metal particles supported onto such substrates are promising potentially catalytically active systems, in which catalytic properties could be tailored by both the nature and size of the metal nanoparticles and also the support material [14].

Wool is a possible natural-fiber substrate for heterogeneous catalysts. Such fibers consist predominantly of a complex protein keratin comprising amino acids with amine, sulfide, carboxylate, and hydroxyl functional groups that have the potential to bind to noble-metal nanoparticles. Wool, particularly New Zealand merino wool, has played an important role in the textile industry over time and is used increasingly as a fine, high-quality natural fiber for luxury apparel [11,15]. Coarser New Zealand crossbred wool is used widely in carpets and upholstery fabrics. However, the potential of wool in heterogeneous-catalysis applications remains largely unexplored. Over recent years, Johnston and coworkers have developed and utilized a method to reduce the salts of noble metals, notably silver and gold, into nanoparticles and bind them to wool fibers to provide a range of noble nanometal–natural fiber composites [12,13]. Their nanogold wool’s, wherein the surface plasmon resonance, colors of gold are used as novel colorants in high-value merino fashion apparel. Their nanogold wool products utilise the surface plasmon resonance effects of the gold nanoparticles as novel colorants in high-value merino fashion apparel. Also their nanosilver wool captures the natural antimicrobial properties of silver and the product is used in antimicrobial textiles. Following on from this, they used a similar approach to prepare nanopalladium–wool fiber composites by functionalizing merino and crossbred wool fibers, respectively, with palladium nanoparticles and characterizing the composite materials produced [16,17,18].

We have extended this work here by studying the catalytic properties of these palladium nanoparticle–wool composites by using them in the liquid-phase hydrogenation of cyclohexene to cyclohexane as a model reaction for this purpose. Being a natural fiber, the chemical and structural characteristics of wool vary somewhat, which, in turn, results in a range of palladium nanoparticles with different sizes and shapes being generated in the wool fibers themselves, which likely affect the catalytic properties of the wool composites. We have endeavored to relate the catalytic performance of palladium nanoparticle–wool composites to the fiber-nanoparticle structure, metal-loading, and nanoparticle sizes of these nanofunctionalized fibers. 

## 2. Materials and Methods 

### 2.1. Materials 

Merino (fine-fiber) and crossbred (coarse-fiber) wool were used as the natural supports of the palladium nanoparticles. The wool was provided by AgResearch Limited, (Hamilton, New Zealand) and all chemicals employed in the synthesis of Pd catalysts were purchased from Sigma-Aldrich (Saint Louis, MO, USA) and used without any additional purification. For the hydrogenation of cyclohexene to cyclohexane, all reactants were analytical-reagent grade and used without further purification. Cyclohexene (99%) was purchased from Fluka (Germany), whereas cyclohexane (99.5%), [n]-decane (99%), and PdCl_2_ were purchased from Sigma-Aldrich (Saint Louis, MO, USA). Palladium on charcoal was purchased from Alfa Aesar (Haverhill, MA, USA) 10.0 wt % Pd on charcoal and dry. Research-grade H_2_ and N_2_ gases (99.98%) were obtained from BOC (North Ryde NSW, Australia). 

### 2.2. Palladium-Wool Nanocatalyst Synthesis

**Figure 1:**
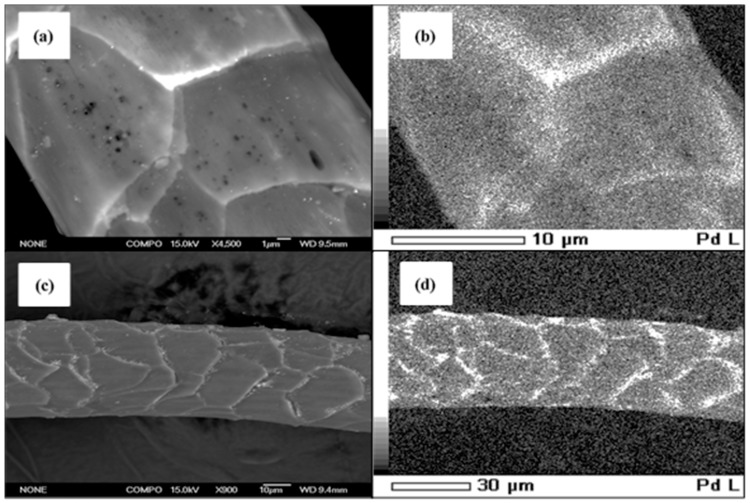
Images of the palladium–merino wool nanocatalysts prepared from a 260 mg L^−1^ Pd^2+^ solution at 100 °C and pH: 3.0 (S1) were taken by (**a**) Backscatter SEM; (**b**) Energy Dispersive X-ray Analysis (EDS) elemental maps for Pd (white dots); (**c**) Backscatter SEM; (**d**) Corresponding EDS elemental maps for Pd (white dots).

Palladium nanoparticles were synthesized by a proprietary method involving the chemical reduction of a palladium-chloride (PdCl_2_) solution onto merino and crossbred wool, wherein the wool acts as the natural support for the palladium nanoparticles formed. The nanoparticle size was controlled by the syntheses conditions and the nanoparticles themselves reside mainly on the external cuticle edges is shown in Figure 1. However, some nanoparticles are formed in the pores of the wool fiber and these are smaller in size due to the small size of the pores within the wool fibers. In this way, the pore volume within the wool fibers exerts some control on the particle size. For this, the wool fibers were soaked in palladium-chloride solutions of concentrations ranging from 6.5 to 260 mg L^−1^ Pd^2+^ at pH 3.0. The pH of these solutions was adjusted using NaOH or HCl solutions, accordingly. Soaking temperatures of 50, 80, and 100 °C from 2 to 24 h were used. Further details were proprietary and are not presented here. Palladium nanoparticles of different sizes were formed from the reduction of Pd^2+^ to Pd^0^ by the coupled oxidation of the disulfide groups in the cystine amino acids of the keratin protein in the wool fibers at these elevated temperatures. This reduction and nanoparticle formation takes place both on the surface of the wool in the vicinity on the cuticles that contain the cystine amino acids in the keratin, and in the fiber pores that are also surrounded by such cystine amino acid—containing keratins (Figure 1). 

**Figure 2:**
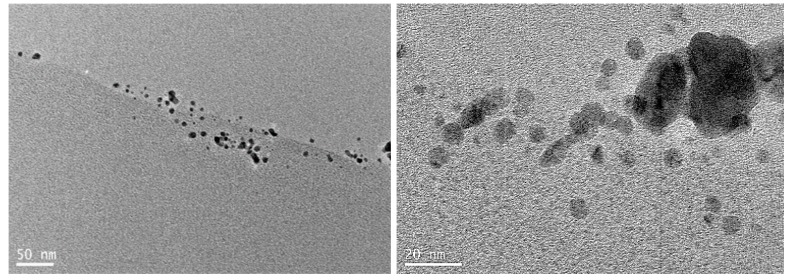
Transmission electron microscope images of a cross section of the palladium–crossbred wool nanocatalysts for S2 with different magnifications showing the Pd particles on the surface of the wool fiber. Similar TEM images were obtained for the other Pd–merino wool nanocatalysts.

The proximity of the dissolved Pd^2+^ to these amino acids is sufficient to affect a redox electron transfer and facilitate Pd-nanoparticle formation. As shown by the transmission electron microscope (TEM) images are shown in (Figure 2) the nanoparticles at the surface are much more varied in size and shape as there is essentially no surface agent to provide any particle size and shape control. However, the nanoparticles formed within the pores are smaller and more uniform due to confined pore volume. At the same time, the palladium nanoparticles were chemically bound to the wool fibers through a Pd-S chemical bond. The nanopalladium catalysts (Pd/wool) samples were labeled with S*n,b* where *n* and *b* are the nanopalladium–wool sample number and the catalysis run number, respectively. Samples S1 to S4 were each provided in 2 batches, which are distinguished by appending 1 or 2 to the S*n* descriptors. S*n*,1 catalysts were provided in the first batch, “the first batch”, whereas *Sn*,2 catalysts were provided as the “the second batch”; meanwhile, S5 and S6 catalysts were provided only as “the second batch”. Because of the natural variation in the properties of wool, particularly the pore sizes, as expected there were slight differences in the size and distribution of the palladium nanoparticles in these various palladium–wool nanocatalysts.

### 2.3. Electron-Microscopy Characterization of Palladium–Wool Nanocatalysts.

A JEOL 6500F field-emission scanning electron microscope (JEOL Ltd, Tokyo, Japan) and a JEOL 2010 transmission electron microscope (JEOL Ltd, Tokyo, Japan) were used to characterize Pd/wool nanocatalysts prepared using merino wool and crossbred wool as the substrates for palladium nanoparticles. For SEM characterization Pd/wool catalyst samples were mounted on aluminum stubs and coated with carbon prior to analysis to reduce charging effects. The TEM samples were prepared by embedding the Pd/wool samples in resin and cutting thin films with a diamond knife, which were the placed on copper grids for TEM analysis.

### 2.4. Apparatus and Hydrogenation Reaction 

The catalytic activity of Pd/wool catalyst samples was studied using the hydrogenation of cyclohexene to cyclohexane reaction carried out in a high-pressure hydrogenation apparatus (Parr 4842, Parr Instrument Company, Moline, IL, USA), which enabled control over temperature, pressure, and stirring rate. The Parra reactor Teflon liner of the reactor was charged with the desired amount of a specific Pd/wool nanocatalysts and approximately 20 g of cyclohexene or a 1:1 mixture of cyclohexene and cyclohexane, flushed 3 times with H_2_ and then pressurized to 400 psi (27.5 bar) with H_2_ gas. The amount of the catalyst used was calculated to keep the mass of palladium nanoparticles constant (1.3 mg Pd) across the series of experiments calculated from the Pd content of the wool in the last column of Table 1. Apart from preliminary work optimizing the conditions, all the catalysis-test reactions were conducted under continuous stirring at 750 rpm at a target temperature of 40 ± 1 °C for 24 and/or 5 h. During each catalytic test run, the mixture of reagents and catalysts was heated over a period of 15 min from ambient temperature to the target temperature, where it was held for the remainder of the reaction. At the end of each test, the reactor was cooled to room temperature before venting any remaining gas. The composition of the product mixture was analyzed by using a Shimadzu GC-2010 gas chromatograph with a FID detector (Schimadzu Corporation, Kyoto, Japan) which was programmed based on Table 2.

In all of the experiments, cyclohexene was present in excess and H_2_ gas was the limiting reagent. Hence, the conversion (*C*%) of reactant to product was calculated as:

*C* = ((ne)*_i_* − (ne)*_f_*)/(nh)*_i_* × 100% (1)

where (ne)*_i_* and (nh)*_i_* are the initial amounts (mol) of cyclohexene and H_2_, respectively, and (ne)*_f_* is the final amount of cyclohexene determined by the GC-FID. The 95% confidence interval for all values of *C*% is ± 6% (2 standard deviations, which is determined essentially entirely by the uncertainly of the volume of gas). This uncertainly constitutes a systematic error and hence substantially smaller (less than 6%) differences between *C*% values may be statistically significant.

## 3. Results and Discussion

Hydrogenation of cyclohexene to cyclohexane was chosen as a model reaction because palladium is known to be an active catalyst for hydrogenation of unsaturated hydrocarbons [19]. It was hypothesized that the catalytic activity of Pd/wool would likely depend on the nature and distribution of the palladium nanoparticles in the Pd/wool-fiber nanocatalysts, the stirring rate, amounts of reactants, gas pressure, and target temperature. Hence, the reaction was investigated in the following manner using these Pd/wool nanocatalysts.

### 3.1. Effect of the Stirring Rate

The stirring rate is an important factor in batch-style heterogeneous catalytic tests since it can affect mass transfer. The results obtained using the S1,1 and S2,1 samples of Pd/wool nanocatalysts with pure cyclohexene are given in Table 3. The test at a high stirring rate of 1100 rpm, usually regarded as near optimal for minimizing mass-transfer limitations, resulted in a conversion to cyclohexane of only 29 ± 6%, probably because of the mechanical damage to the fragile Pd catalyst system. A test using the S1,1 Pd catalyst without stirring gave an even poorer result of ~16%. Intermediate stirring rates led to substantially improved conversions, with the best result of 97% conversion (*C%*: ≈ 97) obtained at 750 rpm. Interestingly, these results showed that the Pd nanoparticles supported on wool displayed the best catalytic activity under moderate rates of stirring due to an effective interaction with the cyclohexene. Hence, it is suggested that, in a later study beyond this work, it should be possible to achieve further improvements in the catalytic performance of these Pd/wool nanocatalysts using flow-through reactor designs, where effective mass transfer can be ensured without mechanical damage of the Pd/wool nanocatalysts due to stirring [20].

### 3.2. Amounts of Reactants 

Ideally, for industrially relevant catalysis-reaction processes it is preferable to use a neat starting material with no solvent to minimize costs and the later need for separation, and also to improve operational safety and minimize environmental risks. However, in the case of the catalytic test reactions discussed here, there were practical limitations that required the use of a solvent. To achieve effective stirring, the volume of liquid in the reactor must be no less than about 25 mL, which corresponds to 20.3 g (0.247 mol) of cyclohexene. The H_2_ gas pressure of 400 psi was chosen, which was within the safe operating range of the reactor and corresponds to mild pressure conditions [21]. By running reactions with 100% consumption of H_2_ using commercial Pd/charcoal catalysts, the amount of H_2_ in the system was found to correspond to ~0.13 mol of H_2_. Consequently, any reaction using more than ~0.13 mol (10.7 g or 13.2 mL) of cyclohexene, will be limited by the amount of H_2_ and calculations based on the initial amount of cyclohexene alone will result in an erroneously low conversion factor. To circumvent this problem while maintaining an appropriate volume of liquid, reactions were performed using ~10 g of cyclohexene (~12.3 mL or 0.122 mol) diluted with ~10 g (12.8 mL) of cyclohexane. This was chosen because, in the case of the hydrogenation of cyclohexene, the cyclohexane product can be used as a solvent to mimic practical industrial conditions. It is also miscible with the reactant and, as the reaction product, it will not interfere with the test reaction.

The effect of variations in the amounts of reactants and products on catalytic performance was studied using the S1,1 catalyst while controlling the stirring rate (750 rpm), pressure (400 psi), and target temperature (40 °C). The data are presented in Table 4. In the second test run, the mass of cyclohexene was reduced to ~10 g, and ~10 g of cyclohexane was added, which resulted in the conversion decreasing from ~97% to ~83%. This difference is significant at the ~97% confidence interval for these single results.

### 3.3. Optimization of H_2_ introduction

Since hydrogen gas was one of the reactants, the effect of the way it was introduced into the reactor was studied using the two protocols listed below while keeping other parameters fixed (~10 g cyclohexane: ~10 g reactant composition, stirring rate: 750 rpm, and a target temperature of 40 °C). 

Protocol 1: The reactor was initially purged with H_2_ three times and then pressurized with H_2_ gas to 400 psi prior to heating to the target temperature. The conversion was 83 ± 6%.

Protocol 2: The reactor was initially purged with N_2_ three times and pressurized to 3 psi with N_2_ gas. After reaching target temperature, the reactor was flushed and pressurized to 400 psi with H_2_ when the temperature reached 40 °C. The conversion was 76 ± 6%.

From the data in Table 5 it appears that the initial purging of the reactor with N_2_ and then introducing H_2_ had no real significant beneficial effect on the conversion of cyclohexene. For further experiments, it was therefore decided to use the simpler procedure of pressuring the reactor with 400 psi of H_2_ from the start.

### 3.4. Temperature 

The target temperature was considered as another parameter for optimization. Reactions were carried out at 20, 30, 40, 50, and 60 °C, while keeping all other parameters fixed (~10 g cyclohexane: ~10 g reactant composition, 750 rpm stirring rate, and initial H_2_ pressure of 400 psi). The conversion increased from 46% to 83% when the temperature was increased from 30 to 40 °C, but then decreased to 66% when the temperature was increased further to 50 °C (Table 6). Meanwhile, the results showed low conversion at 20 and 60 °C. Perhaps at the elevated temperature and pressure, the wool fibers were less robust, leading to mechanical degradation [22].

Despite former research, the result of this part proved that Pd/wool catalysts could be active catalysts even at low temperatures [23,24]. For instance, Lei and coworkers showed hydrogenation of 1-Decene and 1-Octene as reactants in the present of 0.15 mmol g^−1^ Pd/wool nanocatalysts at 70 °C after 24 h reaction time and their yields were about 61% and 54%, respectively [23]. Later, Xin et al. investigated hydrogenation of 5-hexen-2-one at ethanol and stilled water as solvents at 70 °C after 24 h and the yield of product was 99.3% [24].

### 3.5. Comparison of Samples S1–S6

According to previous research, metal loading is also one of the important factors that can affect the catalytic activity of the catalysts [25,26]. In our preliminary studies, catalysts S1 and S2 with 2.6 wt % Pd loadings showed appreciable activity (initially estimated indirectly by monitoring the H_2_ pressure drop) and thus their testing was performed using shorter, 5-h test runs. S3 to S6 (i.e., for both batches), with loadings between 1.6 and 6.4 wt % Pd, gave low conversions (see Table 7 and Table 8), even in 24 h tests. S1,2 from “the second batch” of the catalysts, consistently showed the best performance, with an H_2_ pressure drop of 280 psi within the first hour and *C*: 95 ± 5% over 5 h, a conversion comparable with commercial Pd/C catalyst, albeit over a substantially longer reaction time. Earlier research by Lei and coworkers is not comparable with our results; the synthesized Pd/wool catalysts by them showed low conversion (approximately 10%) of 1-Octene and 1-Decene to alcohol after 5 h at 70 °C [23]. In comparison, S1,1 managed just *C*: 83 ± 6% after a much longer period of 24 h (see Table 6 and Table 7). The difference was that S1,2 tested very much sooner after its synthesis, which suggested that the freshly made catalysts had higher activity.

In Table 8, the results for repeated triplicate tests are shown for some of the samples to illustrate reproducibility. The results were pleasingly generally quite reproducible for the same sample from the same batch. However, the reproducibility between batches was not as strong, even for the same particle size. For example, the activity of S1,2 was very reproducible (*C*: ~95% over 5 h), but significantly greater than for S1,1 (*C*: ~83% over 24 h) even though the samples were produced under the same synthesis conditions. Since every sheep’s wool is somewhat unique, the pore size and protein chemistry will differ slightly. As the wool chemistry to facilitate nanoparticle synthesis and particle size is controlled to some extent by the chemical makeup and pore size of the fibers, it is not surprising that such differences in the catalytic activity between samples are observed. 

It is apparent from Table 7 and Table 8 that factors in addition to metal loading were important in defining the catalytic activity of these Pd/wool nanocatalysts. Hence, it is useful to look at of the how the catalyst morphology (i.e., nanoparticle size and accessibility of the metal nanoparticles to the reagents and the presence of functional groups capable of influencing catalytic activity of metal nanoparticles in the matrix of the support) affected the catalytic performance.

Figure 1 shows the backscattered SEM images and EDS elemental Pd maps for palladium–merino wool and palladium–crossbred wool nanocatalysts prepared using 260 mg L^−1^ Pd^2+^ solution at 100 °C and pH 3.0 for S1. The SEM images (Figure 1a,c) show the Pd particles as very small white dots that are concentrated along the cuticle edges with a very low concentration across the larger cuticle surface of the fiber. The corresponding EDS elemental maps for palladium overlay exactly with the SEM images and confirm these white dots are indeed palladium nanoparticles. The keratin proteins at the cuticle edges have higher levels of sulfur (disulfide linkages) to readily reduce the Pd^2+^ ions and facilitate the preferential binding of the formed Pd nanoparticles to these cuticle edges, as observed here. The distribution of palladium nanoparticles across the fiber surface and the cuticles themselves is essentially the same for both wool-fiber types, merino and crossbred, showing that the fiber itself has little influence on the nature of the distribution of nanoparticles in the particular composite. Figure 2 presents a TEM image of a cross section of the palladium–merino wool nanocatalyst composite, where the palladium nanoparticles are imaged at two different magnifications, similarly showing that these palladium particles are present at and near the surface of the wool fiber. The palladium nanoparticles are typically about 7–10 nm in size, with some larger single particles and agglomerates of the smaller particles up to about 20–40 nm in size. It is likely the smaller Pd nanoparticles act as more effective catalysts than the larger ones. Similar TEM images were obtained for the palladium–merino wool nanocatalysts.

The TEM images show for the S2 sample that the Pd particles range from about 2 to 50 nm in diameter and are again localized on the cuticle surface and within the near-surface regions of the fiber. The nanoparticles have a polydisperse particle-size distribution with larger nanoparticles located on the surface of the fiber and smaller nanoparticles within the fiber itself. It therefore seems that the surface and near-surface location of Pd nanoparticles provided easier accessibility of reactants to the catalytic centers. The smaller nanoparticles would provide more effective catalysis centers. However in Figure 2, it is evident that the smaller particles in S3 to S6 were incorporated further into the fibers and hence these are much less accessible and cannot interact with the reactants as effectively. However, for S1 and S2, the larger nanoparticles were at the surface of the fibers where they were easily accessible to the reactants, but these larger nanoparticles were likely to be less catalytically active. The images show that that not only size and size distributions [27,28,29] but also the locations of the Pd nanoparticles [30,31] affect the catalytic activity of the Pd–wool nanocatalysts. In general, smaller nanoparticle sizes afford better catalytic activity. It is therefore likely that Pd nanoparticles with diameters smaller than 3 nm were more intrinsically effective than larger ones for hydrogenation of organic compounds [28,32]. However, as noted above, since the very small nanoparticles appeared to be contained within the wool fibers, rather than being on the fiber surface, it was difficult for the reactants to access them. It therefore seems likely that the observed catalytic activity for these samples in effect arises from the Pd nanoparticles with diameters of ~5 nm or larger located at or even near the fiber surfaces [27,30,33]. Negligible conversions that were obtained by samples S5,2 and S6,2, which contained Pd nanoparticles with both very small particles sizes and narrow size distributions from 1 to 4 nm, support this hypothesis. The distribution in nanoparticle sizes is a likely cause of the difference in catalytic performance between the various samples prepared and tested here. This is therefore a limiting factor in any potential use of such Pd/wool fiber nanocatalyst materials. 

### 3.6. Comparison between Pd/Wool and Pd/C Nanocatalysts

Palladium particles immobilized on charcoal (Pd/C) are recognized as effective catalysts for hydrogenation of organic compounds. Commercially available Pd/C was used as a benchmark against which to measure the catalytic activity of Pd/wool nanocatalysts for hydrogenation of cyclohexene to cyclohexane. Table 9 shows that 95 ± 5% conversion can be obtained by using Pd/wool nanocatalysts over 5 h with an H_2_ pressure drop of ~45 psi within the first hour. However, under the same conditions, the commercial Pd/C nanocatalysts achieved effectively a 100 ± 5% conversion (the H_2_ in the reactor was essentially all consumed) in just 1 hour. This comparison suggests that the catalytic activity of Pd nanoparticles strongly depends on the type of support as well as the Pd-nanoparticle size [34,35,36]. It appears that the lesser catalytic activity of the Pd/wool nanocatalysts is due to the larger particle sizes of the palladium nanoparticles on the wool-fiber surfaces and the range of particle sizes present. As shown by TEM cross sections of the fibers in Figure 2, the smaller nanoparticles are present largely in the pores within the fiber and are less accessible to the reactants. Hence the potential greater catalytic activity of these smaller palladium nanoparticles is not harnessed. However, the study carried out here shows that such palladium nanoparticle–wool composites do indeed demonstrate catalytic activity in this cyclohexene to cyclohexane reaction and with further refinement could have potential industry applications.

## 4. Conclusions

This study investigated the catalytic activity of Pd/wool nanocatalysts with Pd-nanoparticle loadings from 1.56 to 6.4 wt % whilst controlling the stirring rate, pressure, and target temperature. It is apparent from the study that the catalytic activity of the Pd/wool nanocatalysts is influenced by the few controllable reaction parameters in play here, along with the nature of the wool fibers themselves. Specifically, these relate to the morphology and surface structure of the fiber, which can affect accessible metal loading, as well as the size and size distributions of the Pd nanoparticles during the synthesis process, which were actually accessible to the catalysis reaction (i.e., on the surface of the fiber). Samples S1 and S2, with an average nanoparticle size of around 5 nm and broad size distributions (2–30 nm) proved to be the most active catalysts. S1 and S2 with Pd nanoparticles on merino-wool fibers showed higher activity than those with nanoparticles on crossbred-wool fibers. In this study, Pd/wool nanocatalysts S1 and S2 with 2.6 wt % Pd loadings onto the wool-fiber substrate showed appreciable catalytic activity and thus their testing was performed using relatively short, 5-h test runs. Samples S3 to S6, with loadings of between 1.6 and 6.4 wt % Pd performed poorly, giving low conversions even in 24 h tests. Importantly, S1,2 from “the second batch” catalyst batch showed excellent performance (*C*: 95 ± 6%, comparable with commercial Pd/C nanocatalysts) within 5 h, with an H_2_ pressure drop of 280 psi within the first hour. This, in comparison to performance of S1,1 (*C*: 83 ± 6% after 24 h) suggests that freshly made nanocatalysts based on Pd supported on wool may have a higher activity. Thus, it is apparent that factors in addition to metal loading were important in defining catalytic activity. This study showed that a nanocatalyst’s morphology, notably the accessibility of the metal nanoparticles to the reagents and the presence of functional groups capable of influencing the catalytic activity of metal nanoparticles in the matrix of the support, affects the catalytic performance. Samples S3, S4, S5, and S6 were all below 6% conversion in 24 h. In general, there was no significant difference in the catalytic activity between the catalysts formed using the merino- or crossbred-wool fibers as the substrate. Overall, this work shows that palladium nanoparticles can be formed and bound onto the surface and near-surface of New Zealand fine-merino and coarser-crossbred wool fibers and that the resulting Pd/wool nanocatalysts were effective in catalyzing the reduction of cyclohexene to cyclohexane. The catalytic effectiveness was largely governed by the size and size range of the palladium nanoparticles and the accessibility of the reactants to them. The smaller nanoparticles afford better catalytic activity, which was enhanced further if they were at the surface of the fibers and fully accessible to the reactants. This illustrates a novel use of wool fibers as a substrate for palladium nanoparticles for catalysis reactions. 

## Figures and Tables

**Table 1:** Samples of Pd/wool nanocatalysts with corresponding Pd nanoparticles loadings and the total masses of the nanocatalysts loaded into the reactor for each test.

Pd/Wool Samples	Fabrication Conditions	Load of Pd (Pd/wool: mg g^−1^)	Mass of Pd/Wool (mg)
S1	Merino, pH = 3.0, 100 °C, 2 h, 260 mg L^−1^ Pd^2+^	15.6	50
S2	Crossbred, pH = 3.0, 100 °C, 2 h, 260 mg L^−1^ Pd^2+^	15.6	50
S3	Merino, pH = 3.0, 50 °C, 24 h, 260 mg L^−1^ Pd^2+^	15.6	55
S4	Crossbred, pH = 3.0, 50 °C, 24 h, 260 mg L^−1^ Pd^2+^	15.6	80
S5	Merino, pH = 3.0, 80 °C, 24 h, 6.5 mg L^−1^ Pd^2+^	0.39	20
S6	Crossbred, pH = 3.0, 80 °C, 24 h, 6.5 mg L^−1^ Pd^2+^	0.39	20

**Table 2:** Gas chromatography oven-temperature program: the second column indicates the rate at which the temperature was increased to that indicated by the third column (the temperature prior to the injection was 60 °C). The hold time (last column) indicates the period for which the temperature was maintained.

Step #	Rate (°C/min)	Temperature (°C)	Hold Time (min)
1	-	60.0	0
2	2.0	74.0	0
3	35.0	174.0	0
4	50.0	280.0	5

**Table 3:** Effect of the stirring rate on the conversion of cyclohexene (24 h long tests).

Sample # (Pd/wool)	Stirring Rate (rpm)	Initial Mass of Cyclohexene (g)	Initial Mass of Cyclohexane (g)	*C* (%)
S1,1	1100	20.01	0.00	29
S1,1	750	20.35	0.00	97
S1,1	0.00	20.35	0.00	16

**Table 4:** Conversion% as a function of the initial masses of reactants over 24 h.

Sample# (Pd/wool)	Initial Mass of Cyclohexene (g)	Initial Mass of Cyclohexane (g)	*C* (%)
S1,1	20.35	0.00	97
S1,1	10.16	10.12	83

**Table 5:** The effect of the way H_2_ was introduced during the test on the conversion% over 24 h for sample S1,1.

Mass of Cyclohexene (g)	Mass of Cyclohexane (g)	*P_i_* H_2_ (psi)	*P_i_* N_2_ (psi)	*P* H_2_ (psi) at 40 °C	*C* (%)
10.16	10.12	400	0	0	83
10.14	10.06	0	3	400	76

**Table 6:** The effect of temperature on the conversion % over 24 h.

Sample Number of Pd/Wool	Initial Mass of Cyclohexene (g)	Initial Mass of Cyclohexane (g)	Temperature (°C)	Conversion (%)
S1,1	10.21	10.13	20	10
S1,1	10.01	10.03	30	46
S1,1	10.16	10.12	40	83
S1,1	10.23	10.31	50	66
S1,1	10.07	10.05	60	35

**Table 7:** The conversion % data for nanopalladium–wool catalysts.

Sample# (Pd/Wool)	Mass of Cyclohexene (g)	Mass of Cyclohexane (g)	*C* (%) (First Run)	Time (h)
S1,2	10	10	95	5
S2,2	10	10	78	5
S3,2	10	10	2	24
S4,2	10	10	9	24
S5,2	10	10	1	24
S6,2	10	10	2	24

**Table 8:** Comparison of the catalytic performance of two different batches of palladium–wool nanocatalysts.

Sample # (Pd/Wool)	Initial Mass of Cyclohexene (g)	Initial Mass of Cyclohexane (g)	*C* (%) for First Run	Time (h)
S1,2	10	10	95	5
S1,1	10	10	83	24
S2,2	10	10	78	5
S2,1	10	10	55	24
S3,2	10	10	2	24
S3,1	10	10	3	24
S4,2	10	10	6	24
S4,1	10	10	5	24
S5,2	10	10	1	24
S6,2	10	10	2	24

**Table 9:** Comparison of palladium–wool nanocatalysts with palladium–carbon nanocatalysts.

Sample	Initial Mass of Cyclohexene (g)	Initial Mass of Cyclohexane (g)	*C* (%)	Time (h)
Pd/wool	10	10	95	5
Pd/C	10	10	100	1
